# Functional maturation in preterm infants measured by serial recording of cortical activity

**DOI:** 10.1038/s41598-017-13537-3

**Published:** 2017-10-11

**Authors:** N. J. Stevenson, L. Oberdorfer, N. Koolen, J. M. O’Toole, T. Werther, K. Klebermass-Schrehof, S. Vanhatalo

**Affiliations:** 1 Department of Neurological Sciences, Faculty of Medicine, University of Helsinki, Helsinki, Finland; 2 Department of Pediatrics, Medical University of Vienna, Vienna, Austria; 3 Irish Centre for Fetal and Neonatal Translational Research, University College Cork, Cork, Ireland; 4 Department of Children’s Clinical Neurophysiology, HUS Medical Imaging Center, Helsinki University Central Hospital, Helsinki, Finland

## Abstract

Minimally invasive, automated cot-side tools for monitoring early neurological development can be used to guide individual treatment and benchmark novel interventional studies. We develop an automated estimate of the EEG maturational age (EMA) for application to serial recordings in preterm infants. The EMA estimate was based on a combination of 23 computational features estimated from both the full EEG recording and a period of low EEG activity (46 features in total). The combination function (support vector regression) was trained using 101 serial EEG recordings from 39 preterm infants with a gestational age less than 28 weeks and normal neurodevelopmental outcome at 12 months of age. EEG recordings were performed from 24 to 38 weeks post-menstrual age (PMA). The correlation between the EMA and the clinically determined PMA at the time of EEG recording was 0.936 (95%CI: 0.932–0.976; n = 39). All infants had an increase in EMA between the first and last EEG recording and 57/62 (92%) of repeated measures within an infant had an increasing EMA with PMA of EEG recording. The EMA is a surrogate measure of age that can accurately determine brain maturation in preterm infants.

## Introduction

Premature birth is a significant health problem that affects every tenth live birth and results in half of all admissions to the neonatal intensive care unit (NICU)^1,2^. Mortality and morbidity is increased in neonates born prematurely with neurological deficits persisting over the longer term^3,4^. These deficits result in a 10 to 25-fold increase in annual healthcare costs^5^. The premature brain is highly susceptible to disruption as it undergoes large scale, activity-dependent neuronal wiring during the last trimester^6,7^. Ensuring optimal brain development through dedicated neuro-critical care, therefore, requires effective monitoring of functional brain maturation^8^. The EEG is increasingly being used in this role in the NICU^9^. The major difficulty with the implementation of EEG monitoring is achieving interpretation by the human expert for long periods of time, on demand. This can be effectively overcome using automated analyses.

An important aspect in interpreting the EEG of preterm infants is the use of post-menstrual age (PMA) as a contextual benchmark^10^. An EEG derived measurement of PMA can be used as an objective summary measure of the EEG, used to aid diagnosis and prognosis or incorporated into alternate automated EEG analyses^11–14^. It can also provide support to maturational observations from the visual interpretation of the preterm EEG and be used as benchmark when translating the findings of animal models^15^.

The aim of this study was to apply a measure of EEG maturational age (EMA) to the clinical scenario of tracking functional brain maturation of early preterm infants (<28 weeks gestational age) over several weeks. The cohort of preterm infants used in this study had a normal neurodevelopmental outcome and a wide range of PMA at the time of EEG recording (24 to 38 weeks PMA). The EMA was compared to the clinically determined age (PMA) at the time of EEG recording and key practical aspects of its implementation were assessed in the context of wide scale brain monitoring and as an early outcome proxy for novel interventional studies.

## Results

A total of 1080, 1 h epochs were initially extracted from 43 infants. After the application of artefact detection, 567, 1 h epochs from 101 recordings from 39 infants remained.

**Figure 1:**
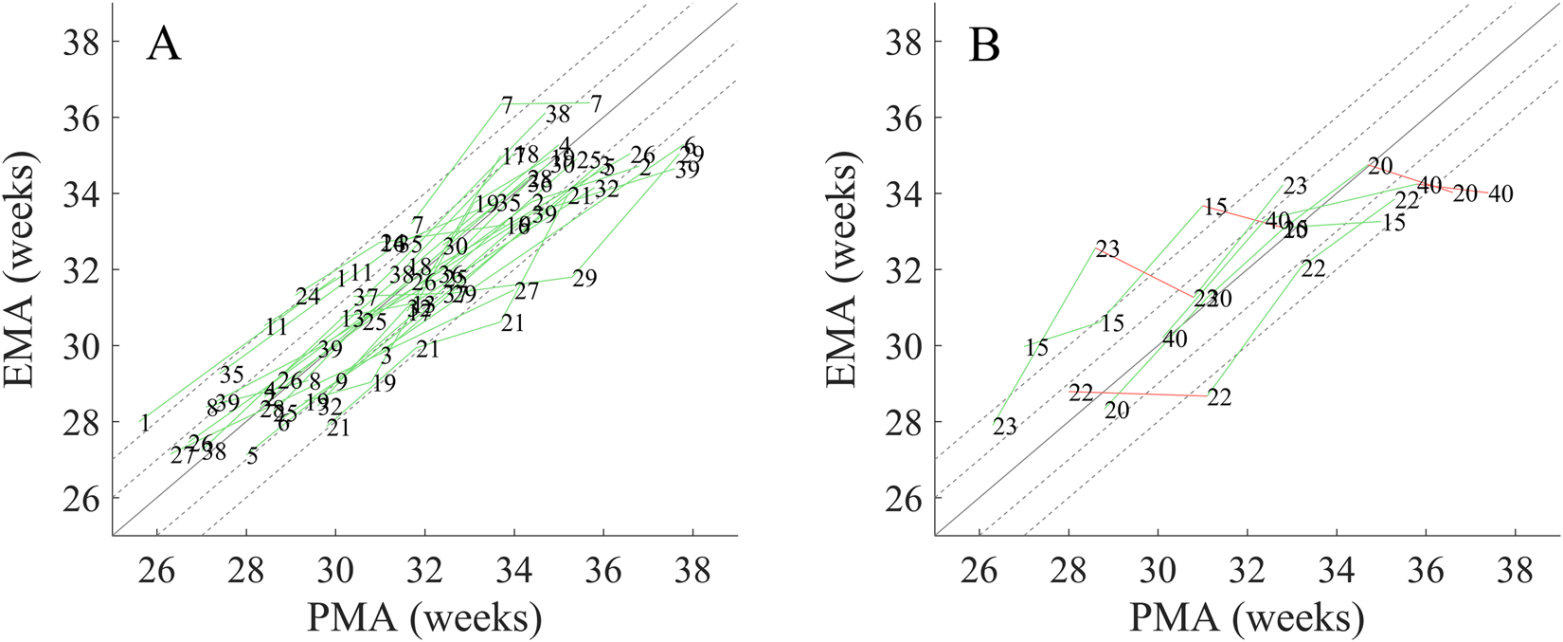
The change in EEG maturational age (EMA) with the post-menstrual age of EEG recording (PMA) in individual infants with normal neurodevelopmental outcome at 12 months of age. (**A**) Infants with constantly increasing EMA with increasing PMA (normal growth) (n = 29). (**B**) Infants with instances of decreasing EMA with increasing PMA (deviant growth) (n = 5). Note that, these instances of deviant growth were due to a single outlier EMA (EMA from a recording with an absolute difference between EMA and PMA greater than 2 weeks). Figures show an EMA estimate based on 46 features with artefact detection and are calculated within a leave-one-infant-out cross-validation on a per recording basis (n = 101). The numbers denote infants and the additional lines track changes in longitudinal recordings: green lines denote an increase in EMA with increasing PMA, while red lines denote a decrease in EMA between consecutive EEG recording despite an increasing PMA. Underlying black lines indicate 0 (solid), 1 (dashed) and 2 (dashed) week differences between the EMA and the PMA.

The EMA based on the full feature set (46 features) was strongly correlated with the PMA at the time of EEG recording. Linear mixed effects modelling resulted in a correlation of 0.936 (95%CI: 0.932–0.976, n = 39). Repeated EEG measures (longitudinal recordings) were available in 34 infants. We found an increasing EMA in all 34 infants (100%) between the first and last EEG recording. In 57/62 recordings (92%) there was an increasing EMA with PMA (Fig. 1A). Five infants had an instance of decreasing EMA with increasing PMA (deviant growth). All instances of deviant growth were due to deviations of the EMA in a single EEG recording by more than two weeks from the age at the time of EEG recording (Fig. 1B).

Several individual features showed a significant correlation with the PMA. The highest correlating features (n = 39) were the 95% percentile of the inter-SAT interval (Low SAT; r = 0.774), RMS of the inter-SAT interval (Low SAT; r = 0.762), 95% percentile of the rEEG (Full; r = 0.751), 95% percentile of the envelope (Full; r = 0.670), total spectral power (Full; r = 0.634), and the number of SATs per hour (Full; r = 0.599). Inter-SAT features were also highly correlated with PMA when estimated on the full recording and amplitude features were also highly correlated with PMA during the low SAT period. All summary measures of relative spectral power and SAT duration had low correlation with PMA (r < 0.5).

**Figure 2:**
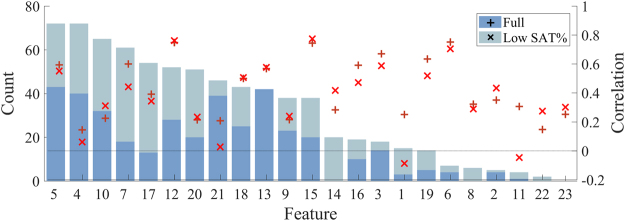
The result of feature selection during the development of the EEG maturational age. The number of times a feature was selected during cross-validation is overlaid with the correlation of each feature with the PMA (cross-validation iterations = 39, feature number = 46); feature numbers align with feature labels given in Fig. 3A. Note that, the features selected most often are not always the features with the highest individual correlation between EMA and PMA. The legend refers to the segment of EEG each feature was estimated on: full denotes full 1 h EEG epoch and low SAT% refers to a segment of EEG with low SAT activity. Similarly, feature correlations are denoted by ‘ + ’ when estimated on the full 1 h epoch and ‘x’ when estimated on the low SAT% segment of the epoch.

The performance of the EMA developed with the incorporation of feature selection is summarised in Table 1. EMA measures outperformed a worst-case estimate (the mean PMA of EEG recording in the dataset). Reducing the number of computational features did not significantly increase the MSE (no FS vs FS; p = 0.912, n = 101). After feature selection, the resultant EMA used a median number of 17 features (IQR: 15–19, n = 39). The five most commonly selected features were the median rEEG, the 5^th^ percentile of the rEEG, 5^th^ percentile of SAT duration, SATs per hour, and the activation synchrony index; these features did not have the highest individual correlations with PMA (see Fig. 2).

**Table 1:** EMA performance with feature selection.

	Mean PMA	SVR	SVR with FS
MSE (days^2^)	438.0	97.3*	108.2*
r	0	0.936	0.940
SD (days)	20.94	9.86	10.30
SE (%)	9.72	4.51	4.75
Bias (days)	0	−0.45	−1.33
% EMA (1 week)	20.8	50.5	43.6
% EMA (2 weeks)	39.6	82.2	85.2

Mean PMA is the worst case/best guess EMA estimate. ^*^Is a significant reduction in the MSE compared to the mean PMA (p < 0.001), SVR is support vector regression, FS is feature selection, PMA is post-menstrual age, EMA is EEG maturational age, SD is the standard deviation and SE is the percentage error and %EMA denotes the percentage of EEG recordings where the EMA was within 1 week and 2 weeks of the PMA and MSE is the mean square error estimated on a per recording basis (n = 101). The adjusted correlation coefficient, r, is estimated from a linear mixed model: the slope of EMA over time was a fixed effect and the intercept was a random effect per infant (n = 39).

Reducing the number of electrodes used to form the bi-polar EEG montage did not significantly affect the accuracy of the EMA (Table 2). A 4-channel EEG montage with frontal polar, temporal and occipital derivations had a MSE that was not significantly different from an 8-channel EEG montage (p = 0.815, n = 101). Two-channel EEG montages containing fronto-temporal or temporo-occipital derivations also had a MSE that was not significantly different from the 8-channel montage (p = 0.427 and p = 0.575, respectively, n = 101).

**Table 2:** EMA performance using reduced channel montages.

4 channel montage	MSE	r	p-value	2 channel montage	MSE	r	p-value
Fp-T, T-O	104.2	0.934	0.815	Fp-T	111.5	0.928	0.427
Fp-C, C-O	112.3	0.898	0.079	T-O	119.7	0.908	0.575
				Fp-C	125.6	0.896	0.025
				C-O	162.6	0.829	0.016
				C-T	172.8	0.789	0.013

The EMA used all features and applied to recordings that passed strict artefact detection. MSE is the means square error, r is the adjusted correlation coefficient (linear mixed model), electrode locations are Fp – frontal polar, C – central, T – temporal, O – occipital, and montages are symmetric across hemispheres. The p-value is estimated using a Wilcoxon signed rank test for paired data, n = 101; the null hypothesis tested is that the MSE was not different from that estimated using an 8-channel EEG montage.

## Discussion

We developed an automated estimator of functional cortical maturation using cot-side EEG recordings from infants with normal neurodevelopmental outcome at 12 months of age. We showed that the EMA correlates with the clinical assessment of PMA of EEG recording over a wide range of ages, all infants had an increasing EMA between their first and last EEG recording, 92% of EEG recordings had an increasing EMA with increasing PMA and 82% of EMA measurements were within 2 weeks of the PMA. Streamlining the EMA measurement by reducing the number of computational features or the number of EEG channels did not significantly affect its performance.

The performance of the EMA is substantial given that there is an inherent error of at least plus or minus one week in the definition of PMA due to variability between conception and the last menstrual period^16^. The remaining variation between the EMA and PMA can be attributed to a combination of errors in training the support vector regression (SVR) and variation in natural biological growth.

Summary measures of EEG amplitude and the temporal characteristics of SATs had the highest individual correlation with PMA. These results align with previous findings that the rEEG and temporal information are correlated with maturation in the EEG^17,18^. Prior studies typically focus on individual features that maximise the correlation with PMA, however, we have shown that when combining features to improve maturational estimates the highest correlating features are not necessarily chosen by the feature selection algorithm. The most notable difference was in the relative beta power (RBP) which has been shown to correlate with PMA in infants with a gestational age less than 32 weeks but was never selected (see Fig. 2; feature 23). This may be due to the dominance of other features representing similar aspects of the EEG, overly age specific correlations that do not necessarily hold over a wider range of PMA or differences in inter-uterine versus extra-uterine maturation^19^. The most commonly selected features were the median and 5^th^ percentile of the rEEG, not the 95^th^ percentile, suggesting that the rEEG width defined by O’Reilly *et al*.^18^ may be improved by incorporating the 50^th^ rather than the 90^th^ percentile. It is interesting that amplitude was the most important maturational feature of the EEG; a measurement that may be considered as underappreciated as a maturational feature in the neonatal EEG literature, although recent work on the analysis of the amplitude integrated EEG (aEEG) supports the usefulness of amplitude for assessing maturation^20–22^. While two rEEG features provide a considerable amount of information to the EMA, they require support from an additional 15 features that measure other aspects of the EEG such as the temporal behaviour of SATs, spectral power and hemispheric synchrony within the EEG.

There are two factors which may challenge the wide scale implementation of EMA monitoring. The first factor is variation in EEG output across NICUs due to differences that may arise from the combination of EEG machine, electrode type, impedance and electrode positioning. Our work shows that an EMA is accurate with the 2-channel bipolar EEG montage that is routinely applied in aEEG monitoring the NICU (the raw EEG signal is readily accessible for automated analysis in aEEG monitors)^23^. The electrode placement must be, however, carefully selected to optimize the susceptibility to artefacts and the information value relating to maturation^24,25^. The second is potential clinical confounders such as neurological condition, non-pharmacological treatments and medication. There is evidence to suggest that medications, particularly sedatives, affect the EEG and should be considered during interpretation^26^. These considerations are also highly relevant when incorporating a variety of neonatal EEG analysis algorithms into neuro-critical care^14,27,28^.

Longitudinal monitoring is a powerful method for assessing human health. In infants, existing methods use measures such as weight, head circumference and clinical assessment of motor function^29,30^. In recent years, analyses have progressed to include measures based functional MRI and DNA methylation in cord blood^31,32^. While it is always difficult to compare studies, the correlation between EMA and the PMA was higher than these methods. Notably, implementation of the EMA in preterm infants has several comparative advantages for wider scale clinical practice. Firstly, it is minimally disruptive and relies on cot-side data that routinely accumulates in the NICU. Secondly, it is a direct measure of the functional development in the preterm brain, which is the target organ in most attempts to improve neonatal care. It also supplements the paradigm of assessing preterm EEG for acute and chronic abnormalities as an additional measurement over a longer time scale^12,33^.

Automated analysis of the EEG can generate a measure of maturation (EMA) that is highly correlated with PMA. The EMA can accurately, and continuously, track the maturation of cortical function in preterm infants over their entire stay in the NICU. The practical significance of this development will ultimately be measured in terms of clinical usefulness. This will be determined in prospective clinical trials which evaluate the added value of such a measure in the individualized neurological care of preterm infants. The EMA also offers an unprecedented opportunity to measure the effects of various treatments and therapies for preterm infants. The EMA as an early outcome measure holds promise for novel interventional studies by expediting their development cycle from several years of follow-up to near real-time assessment.

## Methods

**Figure 3:**
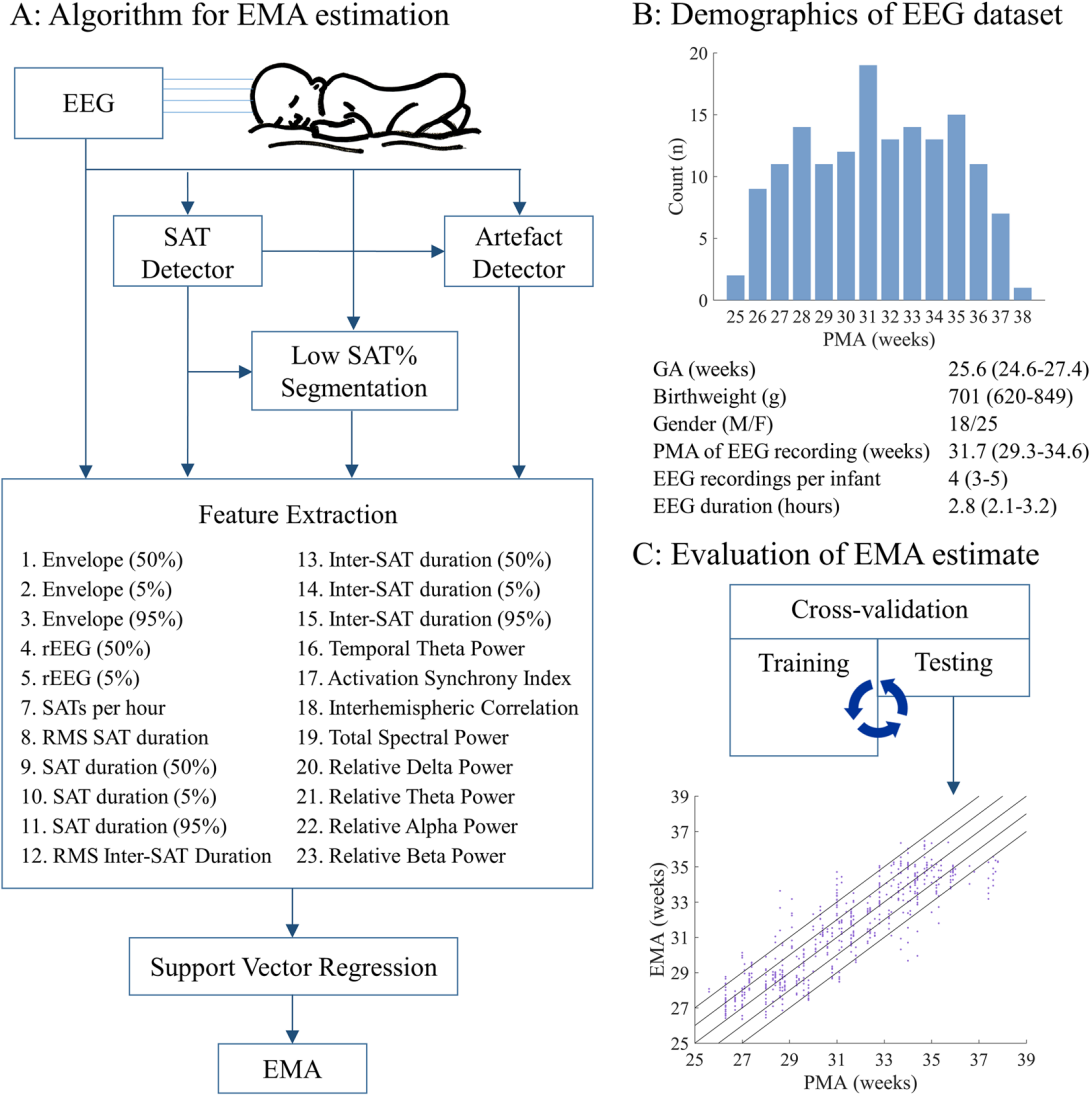
EEG maturational age (EMA) measurement in preterm infants. (**A**) The flow diagram of the EMA algorithm. Percentages in brackets after features refer to the percentile estimated by the feature, for example, envelope (50%) is the 50^th^ percentile or median envelope calculated in the period of interest. SAT is spontaneous activity transient, rEEG is the range EEG, and RMS is the root-mean-square. In this case, delta was 0–3 Hz, theta was 3–8 Hz, alpha was 8–15 Hz and beta was 15–30 Hz. (**B**) The EEG dataset used in this study. The figure shows the distribution of post-menstrual age (PMA) of EEG recordings. The table shows additional demographics of the EEG cohort used in this study - data are summarised as median (interquartile range), except gender which is given as a count. Cohort sample size was 43 infants, 152 EEG recordings and 1080, one hour EEG epochs. (**C**) Evaluating the EMA within a leave-one-subject cross-validation. The dataset was iteratively split into training and test sets and the efficacy of the EMA was assessed by comparing the EMA from the test set to the PMA of EEG recording. The diagonal lines denote errors of plus and minus 0, 1 and 2 weeks.

The overall organisation of this study is shown in Fig. 3. All methods were carried out in accordance with relevant guidelines and regulations.

### Dataset

EEG recordings were collected as a part of a study that recruited infants in the NICU at the Medical University of Vienna, Austria. Multi-channel recordings were acquired at 256 Hz using a Brain Quick/ICU EEG (MicroMed, Treviso, Italy) with a referential (Cz) montage from 9 scalp electrodes located at Fp1, Fp2, C3, C4, T3, T4, O1, and O2. The analysis was performed on a standard bipolar montage: Fp1-C3, C3-O1, Fp1-T3, T3-O1, Fp2-C4, C4-O2, Fp2-T4, T4-O2. Each infant had a cranial ultrasound, once a week, until 34 weeks PMA. Data collection was approved by the local ethics committee (Medical University Vienna, Austria) and the protocol was registered (EK Nr 67/2008). Informed parental consent was obtained for all infants included in the study. The PMA of each infant was defined by the last menstrual period (LMP) of the mother and adjusted using ultrasound measurements in the first trimester if there was significant deviation between LMP and ultrasound analysis.

### Study cohort

The initial study recruited all infants born at less than 28 weeks gestational age who were admitted to the NICU using the following inclusion criteria: the patient was clinically stable, did not receive any brain-acting drugs, and did not have a known neurological morbidity (congenital/chromosomal anomalies and severe perinatal asphyxia). For the inclusion into our present study on EMA validation, we also required that the patient had a normal neurodevelopmental outcome at 12 months of term-corrected age as measured by a Bailey’s II assessment (normal defined as a mental and physical developmental index greater than 85). Out of an initial cohort of 241 EEG recordings from 67 infants, 43 infants (152 EEG recordings) had normal neurodevelopmental outcome, and 16 infants were lost to follow up. Additional demographics of the cohort and the distribution of PMA at the time of EEG recordings are shown in Fig. 3B.

### Calculation of EMA

The EEG signal was initially pre-processed with a band pass filter (low cut-off frequency of 0.5 Hz and a high cut-off frequency of 32 Hz) and then re-sampled to 64 Hz. Each EEG recording was segmented into 1 h long epochs with a 75% (45 minute) overlap. One hour epochs were chosen based on the reported duration of sleep states in preterm infants with the aim of capturing a significant proportion of a full sleep cycle in an epoch^34^. The EEG was then automatically annotated for the presence of spontaneous activity transients (SATs), periods of artefact, and periods of low SAT%^35^. Low SAT% is associated with quiet sleep^36^. The SAT% was defined as the accumulated duration of SAT detections within a 5-minute window (4-minute overlap) expressed as a percentage^34^. The low SAT% period was defined using a threshold (the lower quartile range of the SAT% over time). The estimate of the lower quartile range incorporated an intermediate stage that eliminated SAT% periods with a duration less than 5 minutes.

Several features of amplitude, spatial organization and temporal organization were calculated from the EEG using these annotations. The computational features are listed in Fig. 3A (see also O’Toole *et al*., 2016)^37^. Each feature was estimated per channel and the median value across channels was used. The feature was further summarised using the median across either the entire 1 h EEG epoch or the period of low SAT%, resulting in a total set of 46 features. These features were then combined using a trained SVR to calculate the EMA.

The EEG data was not preselected with any visual criteria, rather a simple automated artefact detection (AD) method was employed to exclude epochs with excessively low or high amplitude. Applying the EEG in a challenging environment such as the NICU results in recordings that are commonly contaminated by a large variety of artefacts. Here, we only consider two types of artefact that are associated with outlying amplitude behaviour^10^. The first type is excessive amplitude artefacts from movements or poor electrode contact. Artefacts were defined using the segmentation provided by a SAT detector^35^. A SAT segment was defined as artefactual if the EEG voltage was greater than 500μV at any time during the SAT. The second type of artefact is excessively low amplitude resulting from electrode shorting (electrodes placed too closely together). These detections affected the entire EEG recording and were performed on a channel by channel basis. Decisions were based on an estimate of the amplitude envelope (magnitude of the analytic associate of the EEG)^37^. Bipolar channels were excluded if the median envelope over time was less than 50% of the median envelope over time and channels. Analysis of referential channels was also used as a basis to exclude bipolar channels derived from the referential electrode. Channels were excluded if the median envelope over time was less than 50% of the median envelope over time and all referential channels and if the 95% percentile of the envelope over the 1 h epoch was less than 25μV. EEG recordings were excluded if any channel had excessively low amplitude or more than 20% of the epoch was contaminated by high amplitude artefact.

### Training and Testing of the EMA

The EMA was trained and tested within a leave-one-infant-out cross-validation. The parameters of the SVR (regularization, ε-insensitive loss and sigma/gamma in the radial basis function) were optimised in an internal 3-fold cross-validation^38^. A feature selection procedure was also performed (backward selection; a complete selection was performed and the number of features selected corresponded to the minimum MSE)^39^. Features were selected within each training iteration using a secondary 10-fold cross-validation and SVR optimisation was incorporated into this training step as a tertiary loop. At each stage of training, the target for optimisation was the minimisation of the MSE between the EMA and the PMA at the time of the EEG recording. SVR was implemented using a MATLAB port (9.0, Mathworks, Natick, MA, USA) of the LIBSVM toolbox^38^.

The ability of the EMA to track development using serial EEG recordings was evaluated using a linear mixed model for repeated measures. The rate of change of EMA over time was assumed to be a fixed effect and the intercept or offset was assumed to be a random effect due to inter-subject variability in maturation and date of conception. The correlation coefficient and the percentage of repeated measures with an increasing EMA with PMA were used as the summary measure of performance. The error between EMA and PMA were also estimated using a range of measures: bias, mean squared error, and standard deviation (expressed as days and percentage) between the EMA and PMA^37^. The association between individual EEG features and PMA was also evaluated using SVR and leave-one-out cross-validation. Values were estimated on the mean value of 1 h epochs extracted from the same EEG recording.

The effect of reducing the number of recording electrodes in the EEG montage was also assessed. This may be needed *ad hoc* as streamlined EEG recording with a lower (typically four) electrode number is part of current NICU practice^23^.

Differences between EMA estimators were tested using a Mann-Whitney Signed Rank test for paired data where a sample was an EEG recording. A p-value less than 0.05 was deemed significant and all tests were two-sided.

### Data Availability

The raw EEG data analysed during the current study are not publicly available due to ethical restrictions. All data generated from the raw EEG data (i.e. the features used) are included in the Supplementary Information files.

## Electronic supplementary material

Dataset 1
